# Stratifying malaria receptivity in Bangladesh using archived rapid diagnostic tests

**DOI:** 10.1186/s12936-020-03418-y

**Published:** 2020-09-23

**Authors:** André Barembaye Sagna, Mohammad Golam Kibria, Shamsun Naher, Shayla Islam, M. M. Aktaruzzaman, Mohammad Shafiul Alam, Cristian Koepfli

**Affiliations:** 1 Department of Biological Sciences, Eck Institute for Global Health, University of Notre Dame, Notre Dame, IN 46556-0369 USA; 2 Infectious Diseases Division, International Centre for Diarrhoeal Disease Research Bangladesh (icddr, b), 68 Shaheed Tajuddin Ahmed Sarani, Mohakhali, Dhaka, 1212 Bangladesh; 3 Communicable Diseases Programme (Malaria), BRAC, BRAC Centre, 75 Mohakhali, Dhaka, 1212 Bangladesh; 4 National Malaria Elimination Program, Communicable Disease Control, Directorate General of Health Services, Dhaka, Bangladesh

**Keywords:** Rapid diagnostic test, *Plasmodium falciparum*, *Plasmodium vivax*, Malaria, Transmission, Surveillance, Salivary biomarker, *Anopheles*

## Abstract

**Background:**

Surveillance of low-density infections and of exposure to vectors is crucial to understand where malaria elimination might be feasible, and where the risk of outbreaks is high. Archived rapid diagnostic tests (RDTs), used by national malaria control and elimination programs for clinical diagnosis, present a valuable, yet rarely used resource for in-depth studies on malaria epidemiology.

**Methods:**

1022 RDTs from two sub-Districts in Bangladesh (Alikadam and Kamalganj) were screened by qPCR for low-density *Plasmodium falciparum* and *Plasmodium vivax* infections, and by ELISA for *Anopheles* salivary gland antibodies as a marker for exposure to vectors.

**Results:**

Concordance between RDT and qPCR was moderate. qPCR detected 31/1022 infections compared to 36/1022 diagnosed by RDT. Exposure to *Anopheles* was significantly higher in Kamalganj despite low transmission, which could be explained by low bed net use.

**Conclusions:**

Archived RDTs present a valuable source of antibodies for serological studies on exposure to vectors. In contrast, the benefit of screening archived RDTs to obtain a better estimate of clinical case numbers is moderate. Kamalganj could be prone to outbreaks.

## Background

In many countries, scaled up malaria control has resulted in reduced morbidity and mortality. Between 2000 and 2015, malaria incidence worldwide fell by 37%, malaria mortality by 60% [1], and an increasing number of countries now aim for malaria elimination. In recent years, however, progress in reducing transmission has stalled, with 228 million cases and 405,000 deaths reported in 2018 [2]. Rapid diagnosis and treatment of symptomatic individuals is critical, but parasite density in many individuals is below the limit of detection of microscopy or rapid diagnostic test (RDT) of around 100 parasites/μL blood. As a result, a large proportion of all infections among febrile individuals escape diagnosis [3, 4]. This limitation also challenges surveillance, that relies on accurate case numbers and prevalence estimates.

In pre-elimination settings, surveillance is crucial to monitor the impact of control interventions and adapt strategies as needed. As alternative to case number routinely reported by health centres, molecular screening can be applied to screen for low-density infections, either among febrile patients, or among the general population to identify asymptomatic infections. Ideally, such screening is accompanied by entomological surveys to measure vector density and composition.

Molecular surveillance in malaria elimination settings across large geographical scales is challenging. Intensive sampling activities are required to obtain sufficient specimens to identify risk factors and determine spatial heterogeneity [5]. As an alternative, specimens routinely collected by control and elimination programmes might be used. In the past two decades, RDTs have become a crucial tool for malaria diagnosis, with over 300 million tests used each year [6]. RDTs are lateral-flow devices that detect parasite proteins in a droplet of blood of approximately 5 µL by immunohistochemistry. Numerous studies have shown that RDTs which have been used by health systems to screen febrile cases could be a useful source of parasite DNA for molecular studies [7–10], and for antibodies for serological surveys to estimate past exposure to malaria parasites [11].

As an alternative to entomological surveys, exposure to *Anopheles* vector bites can be assessed by *Anopheles* biting biomarkers [12]. During blood uptake, the *Anopheles* mosquito injects saliva containing immunogens into the host. Proteomic studies lead to the identification of small salivary proteins specific to *Anopheles* species. The human IgG responses to the gSG6 protein and the gSG6-P1 peptide were validated as serological indicators of exposure to *Anopheles gambiae*, *Anopheles arabiensis* and *Anopheles funestus* [13–15]. The antibody response to *Anopheles* saliva has been showed to gradually increase with increasing intensity of exposure to *An. gambiae* sensu lato (*s.l.*) bites, as assessed by conventional entomological methods [16]. A drop in the anti-saliva IgG response to gSG6-P1 has been observed in children from Angola within a short period of time (6 weeks) after the interruption of exposure by bed net use [17]. The biomarker has been used to accurately evaluate the effectiveness of interventions and changes in transmission intensity in different sites in Africa and Asia [18–22], and seasonal fluctuations of specific IgG antibody levels according to exposure were found [19].

In Bangladesh *Plasmodium falciparum* and *Plasmodium vivax* are endemic along the north-eastern and south-eastern borders. In recent years, a significant decrease of malaria cases and deaths was achieved, with only 10,523 confirmed cases in 2018 compared to 39,719 cases in 2015 (NMEP MIS report). Transmission within Bangladesh is heterogenous. Most cases are reported from the three districts collectively known as Chittagong Hill Tract (CHT) in the south-east of the country [23]. No studies are available that applied PCR screening, and thus the extent of low-density infections is not known.

Each year, over 300,000 RDTs are used by the Bangladesh Malaria Elimination Program (NMEP) to screen febrile patients. qPCR screening was applied to 1022 archived RDTs from two Upazilas (sub-districts) to estimate the proportion of infections missed by routine diagnosis. For the first time, RDTs were used to study heterogeneity in exposure to *Anopheles* mosquitoes using an antibody-based salivary biomarker.

## Methods

### Field specimens

Archived RDTs and routine demographic data collected during diagnosis were obtained from the NMEP from 2 Upazilas in Bangladesh: Alikadam (Bandarban District, n = 522 RDTs, highest transmission in Bangladesh), and Kamalganj (Moulvibazar District, n = 500 RDTs, low transmission) [23, 24]. In Bangladesh, malaria occurs seasonally with the majority of malaria infections in the rainy season from May to October [25]. NMEP workers screen all febrile patients by RDT, and record the result, and data on age, gender, and bed net use using a unique ID, which is also written on the RDT. For the present study, the RDT was matched to the results from the NMEP database (i.e. the RDT result at the time of diagnosis was recorded). The RDTs for this study had been used for diagnosis in August-December 2018 and been stored at ambient temperature until processed in October 2019.

### DNA extraction and qPCR

For initial testing, RDTs were prepared with whole blood spiked with cultured *P. falciparum* at densities of 20,000 and 10,000 parasites/µL. After 10 days of storage at 4 °C, the RDT cassettes were opened, the entire RDT strip was removed, and fragments of the following components were sampled: sample pad and conjugate pad, nitrocellulose strip 1 (between the conjugate pad and the 1st test band), nitrocellulose strip 2 (between the two test bands), nitrocellulose strip 3 (between the 2nd test band and the filter paper), and filter paper. DNA was extracted with the NucleoMag whole blood kit (Macherey–Nagel) and screened by qPCR [26]. *Plasmodium falciparum* DNA was detected in almost all regions of the RDT, with the highest concentration DNA found in the first half of the RDT strip between the sample pad and the first test band. Thus, this part was used for screening of archived RDTs.

DNA was eluted in 25 µL volume, and 4 µL was used for screening for *P. falciparum* using the varATS assay [26] and for *P. vivax* using the *cox1* assay [27]. Both assays target multiple gene copies per parasite.

### Indirect enzyme-linked immunosorbent assay from RDTs

For antibody elution, the second half of the RDT strip was cut into small pieces, placed in 1.5 mL microcentrifuge tubes, and immersed in 250 µl PBS supplemented with 0.05% (v/v) Tween-20 (VWR, USA) (PBS/T). RDT eluates were equivalent to a 1/100 dilution of whole blood or 1/200 of serum (assuming a haematocrit of approximately 50%). Tubes were incubated overnight at 4 °C, after which RDT eluates were separated from solid RDT components and stored at − 20 °C until assayed.

ELISA conditions were standardized as described elsewhere [28]. Testing of serial dilutions of the eluate (1:10, 1:20 and 1:40 in PBS/T) showed optimal performance of the ELISA using a 1:10 dilution.

Nunc-Maxisorp 96-well plates were coated with 100 µL/well of gSG6-P1 peptide (2 μg/mL) diluted in 1 × PBS. Plates were incubated at 37 °C for 2:30 h and blocked with 3 × 300 µL of SuperBlock (TBS) Blocking Buffer. 100 µL of 1:10 diluted sample was added to each well. Plates were incubated overnight at 4 °C, then washed three times and incubated 1:30 h at 37 °C with 100 µL/well of a 1/500 dilution of mouse monoclonal anti-human IgG conjugated with biotin (BD Pharmingen). Peroxidase conjugated extravidine (GE Healthcare Life Sciences) was added at a 1/1000 concentration and incubated 1 h at 37 °C. After four final washes, colorimetric development was carried out using ABTS (2.2-azino-bis (3 ethylbenzthiazoline 6-sulfonic acid) diammonium (Sigma) in 50 mM citrate buffer (pH = 4, containing 0.003% H_2_O_2_), and absorbance (OD) was measured at 405 nm. All samples were tested in duplicate. Each microplate contained in duplicate: positive control, negative control, and blank wells. The positive control was a pool of dried blood spots of people with recent travel to a malaria endemic country, and their result was always above the cut-off for positivity. The negative control was a sample of people from US (n = 2) with no exposure to malaria vectors.

OD normalization and plate-to-plate variation were performed as described elsewhere [29]. Briefly, antibody levels were expressed as the ΔOD value: ΔOD = ODx − ODb, where ODx represents the mean of the technical replicates of each RDT, and ODb the mean of the blank wells. Positive controls of each plate were averaged and divided by the average of the ODx of the positive control for each plate to obtain a normalization factor. Each plate normalization factor was multiplied by the plate sample ΔOD to obtain normalized ΔODs that were used in statistical analyses. The mean ΔOD of negative US controls plus 3 standard deviations (SD) was used to determine cut-off value for responsiveness to the gSG6-P1 peptide. All serological data was analysed qualitatively, i.e. defining individuals as positive or negative based on a cut-off, and quantitatively, i.e. as median of all values (including data below the cut-off). Results are thus reported as seroprevalence, and as median antibody titres.

### Statistical analysis

All data from questionnaires and forms were entered into a Microsoft Access database and statistical analyses were conducted using Graph Prism version 5.05 (Graph Pad Software Inc.). Age, gender, bed net use, and upazila were analysed as risk factors for infection and exposure. The Chi square test was used for the comparison of number of positive samples between sites and age groups. After checking the normality distribution using Kolmogorov–Smirnov and Shapiro–Wilk tests, the non-parametric Mann–Whitney test was used to compare medians of specific IgG Ab levels between two independent groups. The non-parametric Kruskal–Wallis test was used for the comparison of more than two groups. Significance was assigned at p < 0.05.

Analysis by age-groups were considered using two groups: under 18 years-old and 18 years and older owing to the low number of infections recorded in smaller age-groups.

## Results

### Infection status

Table 1 describes the characteristics of the study population. The prevalence of *P. falciparum* and *P. vivax* combined was 3.52% (36/1022) by RDT and 3.3% (31/1022) by qPCR screening of archived RDTs. The majority of infections by RDT (91.7%, 33/36) and qPCR (90.3%, 28/31) were *P. falciparum*; the remaining three were *P. vivax*. All positive RDTs were from Alikadam. RDT positivity was comparable between individuals aged 18 years and older (3.6%, 25/683) and those younger (3.2%, 11/339; p = 0.7).

**Table 1:** Descriptive statistics of participants by study site

Characteristics	Alikadam (n = 522)	Kamalganj (n = 500)
Age, years, median (range)	22 (0.5–70)	27 (2.5–75)
Female gender	46% (240/522)	49.8% (249/500)
LLIN use	98.1% (469/478)	26.4% (132/500)
RDT positivity^a^	6.9% (36/522)	0.0% (0/500)
qPCR positivity^a^	5.7% (30/522)	0.2% (1/500)
Immune responders	9.9% (52/522)	7% (35/500)
Antibody titres, median (range)	0.035 (0.0–2.1)	0.062 (0.0–0.70)

^a^ *P. falciparum* and *P. vivax* combined. Result from time point of diagnostic testing in the field

Concordance between RDT and qPCR was low for *P. falciparum*. Among the 33 positive RDTs, only 20 were positive by qPCR (≥ 1 gene copy/µL eluted DNA). On the other hand, qPCR detected *P. falciparum* DNA from 11 negative archived RDTs. While all RDTs from Kamalganj were negative, qPCR diagnosed 1 low-density *P. falciparum* infection (3.9 copies/µL eluted DNA).

Eight out of eleven RDT-negative/qPCR-positive tests were from adults aged 18 years and older. As the majority of RDTs were from adults, the prevalence of sub-patent infection did not differ between age groups (8/658 in individuals aged 18 years and older, 3/325 in younger, p = 0.671). Likewise, the proportion of sub-patent infections among all infections did not differ (8/31 in individuals aged 18 years and older, 3/31 in younger, p = 0.183). The mean *P. falciparum* density of RDT-negative/qPCR-positive tests (12.9 copies/µL eluted DNA, 95% Confidence Interval (CI 95) 9.4–35.3) was significantly lower than of RDT-positive tests (154.6 copies/µL eluted DNA, CI 95 2.1–311.2; p < 0.0001).

Results were in full concordance for *P. vivax*. All 3 positive RDTs were confirmed by qPCR and no additional infections were detected.

### Exposure to *Anopheles*

**Fig. 1:**
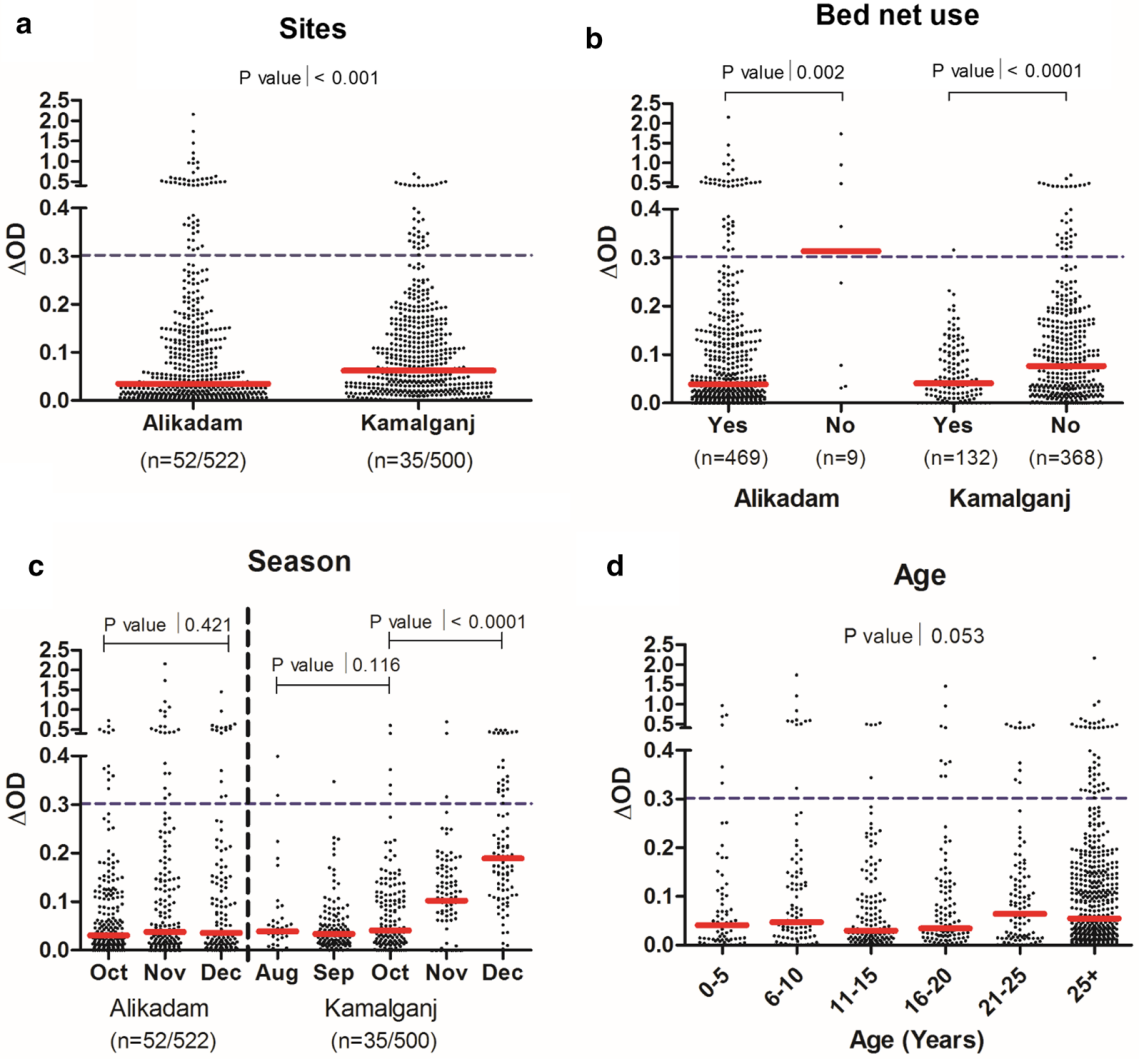
Specific IgG levels to the *Anopheles* gSG6-P1 salivary peptide in Alikadam and Kamalganj, Bangladesh. Black dots indicate individual IgG responses, and bars represent median values in each group. Dotted lines represent the cut-off of a specific Ab response (ΔOD > 0.300). Statistically significant differences between the two (nonparametric Mann–Whitney test) and three or more groups (nonparametric Kruskal–Wallis test) are indicated

The sero-prevalence of specific IgG to *Anopheles* gSG6-P1 salivary peptide did not vary significantly between Alikadam (9.9%, 52/522) and Kamalganj (7.0%, 35/500, p = 0.08, Table 1, Additional file 1: S1). In contrast, the median titres significantly varied between the two study sites and was higher in Kamalganj, the low transmission area (p < 0.0001; Fig. 1a). This result could be explained by lower bed net use in Kamalganj. The rate of mosquito net use 15 days prior the RDT collection was significantly higher in Alikadam (98.1%, 469/478) compared to Kamalganj (26.4%, 132/500, p < 0.0001, Table 1). People who declared having slept under a LLIN (Long-Lasting Insecticidal Net) 15 days prior the RDT collection had significantly lower anti-gSG6-P1 IgG levels compared to those who did not (all p < 0.05; Fig. 1b). In Kamalganj, gSG6-P1 antibody titres remained constant from August to October, and then gradually increased until December (p < 0.0001; Fig. 1c). Titres did not significantly vary from October to December in Alikadam (p = 0.421; Fig. 1c). Median antibody titres did not differ across age groups (p = 0.053; Fig. 1d).

Considering only individuals with antibody titres above the threshold for positivity (n = 87), the median antibody titre was higher in Alikadam (p = 0.0002, Additional file 2: Fig. S2A). This indicates that those that are exposed to *Anopheles* in Alikadam experience higher exposure than exposed individuals in Kamalganj. Not using a bed net remained a significant risk factor for exposure to *Anopheles* (p = 0.0012, Additional file 2: Fig. S2B). Differences across seasons were not significant (p = 0.128, Additional file 2: Fig. S2C), while medians of antibody titres decreased with increasing age (p = 0.0115, Additional file 2: Fig. S2D).

## Discussion

Molecular screening for low density infections and entomological surveys on vector exposure can greatly enhance our understanding of malaria transmission. Sample collection for such surveys spanning large geographical areas is often logistically difficult. This study showed that archived RDTs can present a valuable additional source of specimens for malaria surveillance. While few additional infections were detected, pronounced differences in vector exposure were observed between seasons, and higher exposure was shown in individuals not using a bed net even in a low transmission setting.

As observed in other studies [8], screening of archived RDTs by qPCR yielded few additional infections. In contrast, comparing RDT for diagnosis of febrile patients to qPCR using properly stored blood samples, RDT sensitivity is often below 50% [4]. The low volume of blood applied to the RDT and further dilution during DNA extraction, combined with long storage at ambient temperature resulted in markedly reduced sensitivity by qPCR. DNA degradation can also explain the high number of positive RDTs that were negative by qPCR, along with other factors such as persistence of the antigen after parasite clearance or cross-reactivity of the antibody bound to the RDT with non-*Plasmodium* antigens [30]. In summary, the benefit of qPCR-screening of archived RDTs to obtain a better picture of case numbers was moderate.

Studying vector density as a surrogate marker for the risk of malaria re-emergence is costly [5]. Measuring exposure to *Anopheles* vectors through salivary gland antibody biomarkers presents a cost-efficient alternative, in particular when archived RDTs are analysed.

Exposure to *Anopheles* mosquitoes was higher in Kamalganj where transmission is low, compared to Alikadam, a high transmission area. This result can be explained by much lower bed net use. Only a quarter of patients in Kamalganj reported sleeping under a bed net, compared to nearly all in Alikadam. Antibody titres in individuals from Kamalganj using a bed net did not differ from those in Alikadam. Overall, the results indicate frequent exposure to vectors in Kamalganj despite a low number of clinical malaria cases. Detailed entomological surveys will be required to accurately quantify vector density and behavior, such as the proportion of indoor biting that could be prevented by higher bed net use. The exposure to *Anopheles* increased significantly from October to December in Kamalganj, likely representing seasonal changes in transmission potential [31]. Given the high human-vector contact in Kamalganj in November and December, this site could be prone to outbreaks. The risk is particularly high as *hrp2* deletions have been reported from this area, hampering diagnosis by RDT [32].

The seropositivity values determined in this study cannot be compared directly to values measured in population surveys elsewhere [18–21]. First, samples included only febrile patients. Their exposure might not be representative for the entire population. Second, the volume of blood on the RDT is minimal, and storage at ambient temperature resulted in degradation of antibodies. This was evident by low median IgG levels of anti-gSG6-P1. The technical error of the measurements was probably increased because of the storage conditions. Analysing a larger blood volume stored in a freezer would likely yield higher antibody titres. Future studies comparing RDT and whole blood stored under different conditions will be necessary to quantify the impact of storage. Nevertheless, as storage conditions were the same for all RDTs, results obtained in this study from different sites or across age groups can be compared among each other.

## Conclusions

This study demonstrated the usefulness of archived RDTs for quantifying human-vector contact, and in general for serological surveys in elimination settings. While the low number of infections in Kamalganj precluded analysis of the benefits of bed net usage, the serological data clearly showed that exposure to vectors is high among those not using bed nets. The presence of a sub-patent *P. falciparum* infection detected by qPCR, and sporadic reports of clinical cases highlight the potential for outbreaks. Thus, malaria prevention interventions need to be maintained in low transmission settings in Bangladesh.

## Supplementary information

**Additional file 1.** Serological database.

**Additional file 2: Figure S2.** Specific IgG levels to the *Anopheles* gSG6-P1 salivary peptide for seropositive individuals only (n = 87).

## Data Availability

All data is available within the manuscript and in Additional file 1: S1.
