# Supplementary material for: Stratifying malaria receptivity in Bangladesh using archived rapid diagnostic tests
Source: Malar J. 2020 Sep 23;19:345. doi: 10.1186/s12936-020-03418-y (PMC7513508; doi:10.1186/s12936-020-03418-y)
Supplement: Supplementary file 2 — Additional file 2: Figure S2. Specific IgG levels to the Anopheles gSG6-P1 salivary peptide for seropositive individuals only (n = 87). [file 12936_2020_3418_MOESM2_ESM.docx]

**Stratifying malaria receptivity in Bangladesh using archived rapid diagnostic tests**

**Supplementary Figure S2: Specific IgG levels to the *Anopheles* gSG6-P1 salivary peptide for seropositive individuals only (n=87).**

Black dots indicate individual IgG responses, and bars represent median values in each group. Dotted lines represent the cut-off of a speciﬁc Ab response (ΔOD > 0.300). Statistically significant differences between the two (nonparametric Mann-Whitney test) and three or more groups (nonparametric Kruskal Wallis test) are indicated.
